# Leveraging the digital economy for enhanced digital service trade exports: Lessons from China

**DOI:** 10.1371/journal.pone.0333798

**Published:** 2025-10-30

**Authors:** Xiaoqing Ma, Yunke Huo, Tingting Zhu

**Affiliations:** 1 School of Economics and Management, Ma’anshan University, Ma’anshan, China; 2 School of Business, Anhui University of Technology, Ma’anshan, China; Macquarie University, AUSTRALIA

## Abstract

This study explores the transformative role of digital economy development on digital service trade exports, using China as a case study, to elucidate trends and lessons that may be applicable to other nations seeking to leverage digital advancements for economic growth in an increasingly interconnected global market. Analyzing panel data from 105 global economies between 2013 and 2021 with a trade gravity model, supplemented by robustness and heterogeneity tests, we demonstrate that the development level of the digital economy in trading nations significantly enhances China’s digital service trade exports, particularly impacting middle- and high-income countries. The sectors most affected include intellectual property and cultural services. Our findings reveal a notable positive correlation between digital economy indices and digital service export volumes, suggesting that advancements in digital infrastructure, market dynamics, and governance in partner countries facilitate this increase. Geographical distance and economic systems also play crucial roles, influencing trade costs and compatibility, respectively. These insights guide targeted policy recommendations to enhance digital service trade, emphasizing the need for strategic international cooperation and infrastructure investment to harness digital globalization’s full potential.

## 1. Introduction

The rapid emergence of digital technologies—including cloud computing, big data, artificial intelligence, and the Internet of Things—has profoundly reshaped the global economic landscape. These innovations have elevated the digital economy into a central force driving socioeconomic development. In particular, digitalization has redefined international trade, transforming formerly non-tradable services into tradable ones through digital channels [1–3]. As a result, digital services trade has become a strategic priority for economies seeking diversification and enhanced global competitiveness [4,5]. This trend is evident worldwide. Nations across development stages are increasingly embedding digital services into their trade strategies [6–8]. China exemplifies this shift: in 2021, despite a volatile global economy, its digital services exports reached USD 199.7 billion, comprising 67% of total services exports—a clear testament to the transformative role of digital technologies in trade performance.

Originally defined through the lens of internet-driven innovation—including e-commerce and B2C platforms—the concept of the digital economy has since evolved [9,10]. Bukht and Heeks (2018) [11] proposed a layered framework: a core of information and communication technologies (ICT); an intermediate layer supported by digital tools; and an outer layer of economic activities influenced by digitalization. Recent initiatives have aimed to quantify digital economic development through composite indices such as the EU’s Digital Economy and Society Index (DESI), the Network Readiness Index (NRI), and China’s Global Digital Economy Development Index. Complementary approaches—such as those of Sidorov & Senchenko (2020) [12] and Chao et al. (2023) [13]—conceptualize digital capacity through dimensions like informatization and intelligence.

The relationship between the digital economy and trade has drawn considerable academic interest. Studies consistently show that digitalization influences trade structures, enhances efficiency, and reduces costs [14–17]. For instance, Bunje et al. (2022) [18] found that digital development amplifies the impact of financial deepening on trade outcomes across African economies. Yuan and Han (2023) [19] reported that digitalization has accelerated China’s shift from processing to ordinary trade, while demonstrated that digital development in importing countries enhances the structural upgrading of exporters’ service trade. Additional work underscores the role of digital technologies in lowering trade barriers and boosting export efficiency [15]. Sector-specific research, particularly on services trade, highlights similar trends. For example, Tao and Zhang (2022) [20] showed that the digital economy significantly bolsters services trade, especially in high-income regions, while Ge and Zhang (2023) [21] found that China’s digital development positively affects both the volume and composition of its services exports.

Despite these advances, the impact of the digital economy on digital services trade—the cross-border exchange of services delivered through digital means—remains underexplored [22–25]. A major barrier is the lack of a unified conceptual and statistical framework, which has impeded systematic research [25,26]. The term “digital services trade” was first introduced in a 2012 report by the U.S. Bureau of Economic Analysis (BEA), and later refined by the OECD (2019), which categorized it along three dimensions: delivery modes, product types, and transaction agents. Definitions now range from narrow (limited to digital service provision) to broad (including digital trade in goods). In China’s case, empirical evidence suggests that the narrow definition—focusing on services transformed by digital technologies—offers the most accurate reflection of its trade dynamics [27].

Current efforts to measure digital trade remain fragmented. Frameworks vary by institutional origin: UNCTAD and the U.S. Department of Commerce adopt a single-subject, single-dimension model, while the OECD, WTO, and IMF employ multi-subject, multi-dimensional approaches. The former is suited to narrower conceptualizations and aligns with China’s prevailing trade structure, enabling more consistent international benchmarking and policy formulation. It also complements established paradigms of e-commerce, with digital services trade focused on intangibles and e-commerce centred on physical goods [28].

This study contributes to the literature in two principal ways. First, it clarifies the conceptual boundaries and measurement architecture of China’s digital services trade, adopting the UNCTAD classification and the single-subject, single-dimension framework to reflect sector-specific realities. Second, it applies a gravity model of trade to elucidate how varying levels of digital economy development across countries shape China’s digital services exports. The model also investigates heterogeneity across subsectors—including intellectual property, cultural services, and telecommunications—thus offering policy-relevant insights into optimizing trade structure and competitiveness.

## 2. Model setting and variable selection

### 2.1 Variable selection and data sources

(1) Explained Variable: China’s Digital Service Trade Export (EX)

Both the United Nations Conference on Trade and Development (UNCTAD) and the U.S. Department of Commerce (USDOC) have prioritized the development of methodologies to quantify narrowly defined digital service trade [29]. Central to these efforts is a “single-transaction-object, single-dimension” framework, which identifies digital service trade as the subset of EBOPS-classified services that are delivered via digital means. Building on the approaches of and Gao et al. (2023) [30], this study estimates China’s exports of digital services. We begin by establishing proxy indicators for the export digital integration ratio, which distinguishes between digitally integrated services (those actually delivered or ordered digitally) and digitally integrable services (those potentially deliverable or orderable through digital means).

Following Gao et al. (2023) [30], we draw on data from the Integration of Information and Industrialization Public Service Platform—maintained by the National Industrial Information Security Development Research Center, with coverage of 10,262 service enterprises from 2018 to 2020. From this source, we derive four proxy indicators: (1) total procurement value via the integration platform; (2) enterprise-level online procurement; (3) total sales value; and (4) online sales value. As firms do not report domestic and cross-border sales separately, nor disaggregate goods from services in the dataset, we assume digitalization rates are equivalent across these dimensions. Consequently, the ratio of online to total sales is used as a proxy for both procurement and export digital integration.

Next, platform-based service firms are categorized into six UNCTAD-aligned service sectors: insurance and pension services; financial services; telecommunications, computer and information services; personal, cultural and recreational services; intellectual property services; and other business services. The digital integration ratio for each subsector is calculated as


$$F_{a}^{Ex}=\left(\sum {\mathrm{\backslash nolimits}}_{i=1}^{n}\frac{{Ex}_{i}^{\text{0}}}{{Ex}_{i}^{p}}\right)\;/n$$ (1)

Where, $F_{a}^{Ex}$denotes the digital integration ratio for subsector service exports; ${Ex}_{i}^{\text{0}}$ and ${Ex}_{i}^{p}$ represent the online and total sales, respectively, of sample firm *i*, and *n* is the number of firms in the subsector.

Finally, we apply these ratios to sectoral export values drawn from the WTO-OECD Balanced Trade in Services (BATIS) database. Multiplying each sector’s export volume by its corresponding digital integration ratio and aggregating across all sectors yields an estimate of China’s total exports of digitally delivered services.

(2) Core Explanatory Variable: Digital Economy Development Level (DIG)

In assessing the development level of the digital economy, we draw upon the “Global Digital Economy Development Index Report (TIMG 2023)” issued by the Chinese Academy of Social Sciences on May 30, 2023. This comprehensive report adopts an international perspective and evaluates 106 economies worldwide between 2013 and 2021. The assessment is based on four key dimensions: digital technology, digital infrastructure, digital market, and digital governance. Notably, the report provides both the overall index for global digital economy development and individual rankings for each of the four sub-indexes. By synthesizing existing digital economy indices, it contributes to the formulation of a comprehensive evaluation framework for digital economy development. Data on the digital economy levels of selected trading partner countries during the sample period, along with explanatory notes, are provided in **Table C1**.

### 2.2 Model setting

Following the methodological frameworks proposed by Wu and Wu (2020) [31], Zhang et al. (2019) [32], and Luo et al. (2023) [16], we specify the trade gravity model as follows:


$${\;lnEX}_{i,t}=a_{\text{0}}\;{+\;a}_{\text{1}}{lnDIG}_{it}+a_{\text{2}}{lnGDP}_{it}\;{+\;a_{\text{3}}\ln{POP}_{it}+a}_{\text{4}}{lnDIS}_{it}+a_{\text{5}}{lnEID}_{it}+a_{\text{6}}{lnBA}_{it}\;{+\;a}_{\text{7}}{lnINTER}_{it}+\eta_{i}+\mu_{t}+\varepsilon_{it}\;$$ (2)

Where,$\;{EX}_{i\;}$ denotes China’s digital service trade exports to partner country *i* in year *t,* and ${DIG}_{i}$ represents the digital economy development index of country *i* in the same year*;* higher index values indicate more advanced digital economies*.* The term $a_{\text{0}}\;$is a constant, while $a_{\text{1}}$*-*$a_{\text{8}}\;$denotes the estimated coefficient. $\eta_{i}$ and $\mu_{t}$ represent country and year fixed effects, respectively. Time fixed effects are included to account for macroeconomic shocks and global trends that uniformly affect all countries, such as exchange rate fluctuations (e.g., movements in the US dollar), international economic cycles, technological change, and shifts in global trade policy. Country fixed effects capture time-invariant characteristics unique to each partner country, including geographic features, cultural norms, and institutional or policy frameworks that persist over time. The term $\varepsilon_{i,t}$ captures the random error, santerm. In line with Luo et al. (2023) [16] and Chao et al. (2023) [13], we include additional control variables—namely ${GDP}_{i},\;{DIS}_{i}$, ${EID}_{i}$,${\;BA}_{i\;}\;and\;{INTER}_{i}$—as detailed in **Table 1**.

**Table 1:** Variable description and data sources.

	Descriptions	Sources
${EX}_{it}$	China’s digital service trade exports to the trading country *i.*	WTO-OECD
${DIG}_{it}$	The digital economic development of the trading country *i*.	TIMG 2023
${GDP}_{it}$	The current US dollar value of the GDP for trading country *i*	World Bank WDI database
${POP}_{it}$	The population size of trading country *j* during year *t.*	World Bank WDI database
${DIS}_{i}$	The distance between China and trading country *i.*	Baidu Map Ranging
${EID}_{it}$	The economic system differences between China and trading countries *i*.	American Heritage Foundation
${BA}_{it}$	The number of fixed broadband access users in trading country *i*.	World Bank WDI database
${INTER}_{it}$	The percentage of interconnected users in trading country *i*.	World Bank WDI database

To facilitate a comprehensive understanding of the dataset prior to conducting empirical analysis, we performed descriptive statistical analysis on the variables. **Table 2** presents key statistical measures for each variable, including the number of observations, mean, standard deviation, minimum, and maximum values. The mean value of digital services exports (EX, in million USD) is 950.67, with a standard deviation of 2,287.98, indicating substantial variability across countries. The maximum and minimum values—27,213.48 and 0.37, respectively—underscore the pronounced disparity in digital services trade, reflecting the uneven development of digital service capabilities among nations. While variation in digital economy levels is also observed, the extent of disparity is comparatively smaller than that of digital services trade.

**Table 2:** Descriptive statistics of key variables.

	Observations (Obs.)	Mean	Standard Deviation (Std. Dev.)	Minimum (Min)	Maximum (Max)
*EX*	945	950.67	2287.98	0.37	27213.48
*DIG*	945	50.58	18.05	10.67	95.28
*GDP*	945	639	2040	4.05	23300
*DIS*	945	8846.26	4050.77	955.65	19297.47
*POP*	945	499	1400	0.688	1410
*EID*	945	59.3	12.5	3.0	83.9
*BA*	945	17.75	13.77	0.01	48.76
*INTER*	945	63.37	25.80	4.40	100.00

Notes: EX, GDP, DIS, POP, and BA are measured in millions of US dollars, hundreds of millions of US dollars, kilometers, millions of people, and millions of users, respectively. EID refers to the Economic Freedom Index, published by The Heritage Foundation (2023), with scores ranging from 0 to 100; higher scores indicate greater economic liberalization. DIG is obtained from the Global Digital Economy Development Index Report. It is a unitless index ranging from 0 to 100, where higher values reflect more advanced levels of digital economic development. INTER denotes internet penetration, expressed as a ratio; higher values indicate broader internet coverage within a country.

## 3. Empirical studies

### 3.1 Model test

Before estimating Equation (2), diagnostic tests were performed to assess potential correlation, multicollinearity, and non-stationarity among the variables, aiming to reduce the risk of biased estimates and spurious regression. As shown in **Table A1 and Table A2**, digital economic development correlates positively with Chinese digital services trade exports (statistically significant at p < 0.05), indicating that higher digital readiness among trading partners is associated with increased export volumes. All explanatory variables exhibited a mean variance inflation factor (VIF) of 6.47 (**Table A2**), well below the conventional multicollinearity threshold of 10, confirming negligible collinearity concerns. Levin–Lin–Chu (LLC) panel unit root tests revealed all variables to be stationary at the 1% significance level (detailed results in **Appendix A in** S1 File). The time-invariant variable DIS was excluded from this analysis, as unit root tests assess time-series stationarity by design.

### 3.2 Analysis of empirical results

To examine the determinants of China’s digital service trade exports, we apply a gravity model of trade, accounting for both global shocks—such as economic crises, technological disruptions, dollar deflation, and shifts in tariff policy—and country-specific characteristics, including geography, cultural traditions, and long-term policy orientations. Following the empirical strategies of Wu and Wu (2020) [31], Luo et al. (2023) [16], and Chao et al. (2023) [13], we employ a two-way fixed effects panel model (Equation 2) to obtain baseline coefficient estimates. Robustness checks are conducted via F-tests, Lagrange Multiplier (LM) tests, and Hausman tests, comparing outcomes across fixed effects, random effects, and pooled panel specifications. Full test results are reported in **Appendix B** in S1 File.

To address potential heteroskedasticity and zero trade flows—common issues in trade data—we additionally adopt the Poisson pseudo-maximum likelihood (PPML) estimator, as advocated by Olivero and Yotov (2012) [33], Anderson (2014) [34], and Larch and Yotov (2024) [35]. **Table 3** presents results under three model specifications: Model 1 (no controls), Model 2 (with control variables), and Model 3 (with both controls and fixed effects). Robust standard errors are reported in parentheses (clustered at the country level). Across all models, the digital economy index of China’s trading partners exerts a statistically significant and positive influence on its digital service exports, supporting the hypothesis that greater digital integration lowers trade frictions and reduces marginal costs [31].

**Table 3:** Regression results of the gravity model Dependent variable: China’s Digital Service Export.

	Model 1	Model 2	Model 3
${lnDIG}_{it\;}$	1.6922^***^	1.1549^***^	1.2255^***^
	(0.0903)	(0.0962)	(0.0822)
${lnGDP}_{it}$		0.1235^***^	0.0888^***^
		(0.0074)	(0.0130)
${lnPOP}_{it}$		0.0910^***^	0.0749***
		(0.0100)	(0.0093)
${lnDIS}_{i}$		0.0283*	0.0208*
		(0.0105)	(0.0097)
${lnEID}_{it}$		−0.0061	−0.0119
		(0.0199)	(0.0170)
${lnBA}_{it}$		0.1439^***^	0.0584^***^
		(0.0211)	(0.0143)
${lnINTER}_{it}$		−2.3106	−2.5421
		(1.2595)	(1.3913)
Country fixed effects	No	No	Yes
Time fixed effects	No	No	Yes
*N*	945	945	945
r^2	0.6846	0.8020	0.8125

Note: Robust standard errors, clustered at the country level, are reported in parentheses. *p < 0.1, **p < 0.05, ***p < 0.01.

Partner GDP and population size are also positively associated with China’s digital service exports, indicating that economic scale and market size remain key drivers of demand in the digital services sector. Notably, geographical distance exhibits a counterintuitive positive association with exports. This result diverges from traditional trade models, where distance typically proxies for cost and risk [36]. In digital service trade, however, spatial separation imposes minimal constraints, as transactions are decoupled from physical logistics. Notably, our robustness analysis did not include results from generalized method of moments (GMM) estimation or models incorporating lagged values of key explanatory variables; additionally, geographical distance (DIS, as abbreviated herein) showed no significant association with the primary outcome.

Variables such as differences in economic systems and internet penetration rates (measured as internet users per capita) do not significantly influence trade volumes. This may reflect the diminishing relevance of systemic divergence in an increasingly interconnected global economy, as deeper global market integration and the ongoing liberalization of China’s economy have substantially reduced trade barriers rooted in systemic economic differences relative to the previous century. In contrast, fixed broadband subscriptions per 100 inhabitants show a robust positive effect, underscoring the importance of physical infrastructure in facilitating digital trade.

A Wald test of joint significance yields a test statistic of 2025.63 (p < 0.0001), rejecting the null hypothesis that the model’s coefficients are jointly zero. This indicates that, taken together, the explanatory variables exert a statistically significant impact on China’s digital service trade exports, affirming the relevance and coherence of the proposed framework.

### 3.3 Robustness test

Given the absence of a uniform standard for defining the digital economy, its development level can be measured through various dimensions to account for potential differences in the impact on China’s service trade exports. Following Luo et al. (2023) [16] and Wang et al. (2021) [37], we use the four sub-indices of the Global Digital Economy Index (TIMG), published by the Chinese Academy of Social Sciences in 2023: digital technology (Technology), digital infrastructure (Infrastructure), digital market (Market), and digital governance (Governance). The robustness of the empirical results is tested using these sub-indices. Following van der Marel and Ferracane (2021) [38], our analysis also incorporates one-period (*lndDIG_lag1*) and two-period (*lnDIG_lag2*) lagged values of the core explanatory variable. For robustness, these replacements are estimated using the same PPML approach as in the baseline regression. Doing so can both test for endogeneity issues and further test the reliability of the regression estimates in this study.

Because the Generalized Method of Moments (GMM) effectively addresses endogeneity in dynamic panels, accommodates short panel data, controls for unobserved individual heterogeneity, and improves estimation efficiency [23,39], we employ system GMM to further assess the robustness of our findings.

**Table 4** indicates that all four alternative variables exert a significant positive influence on China’s digital service trade. The GMM estimation reported in model (1) of **Table 5**, along with the lagged core explanatory variables in models (2) and (3), further supports the robustness of the regression results, reinforcing the stability of the findings.

**Table 4:** Robustness checks using sub-indices of digital economy development.

	Digital technique (1)	Digital infrastructure (2)	Digital market (3)	Digital governance (4)
*lnTEC*	0.0524***			
	(0.0110)			
*lnINF*		0.0964***		
		(0.0337)		
*lnMAR*			0.1965***	
			(0.0406)	
*lnGOV*				0.0231**
				(0.0125)
*lnGDP*	0.1073***	0.0928***	0.0829***	0.1008***
	(0.0229)	(0.0211)	(0.0212)	(0.0230)
*lnPOP*	0.117**	0.147***	0.067	0.109**
	(0.053)	(0.048)	(0.049)	(0.054)
*lnDIS*	0.0276*	0.0289*	0.0173	0.0278*
	(0.0155)	(0.0151)	(0.0132)	(0.0155)
*lnEDI*	−0.0057	−0.0100	−0.0123	−0.0064
	(0.0160)	(0.0161)	(0.0153)	(0.0161)
*lnBA*	0.0696**	0.0709***	0.0406*	0.0694***
	(0.0274)	(0.0269)	(0.0234)	(0.0269)
*lnINTER*	−2.3907	−2.4584	−2.5767	−2.6061
	(2.9581)	(2.8660)	(2.7668)	(2.8921)
Country fixed effects	Yes	Yes	Yes	Yes
Time fixed effects	Yes	Yes	Yes	Yes
N	945	945	945	942
R^2	0.5724	0.5725	0.5726	0.5727

Note: Robust standard errors, clustered at the country level, are reported in parentheses. *p < 0.1, **p < 0.05, ***p < 0.01.

**Table 5:** Robustness test results for dynamic GMM estimates and lagged core explanatory variables Dependent variable: China’s Digital Service Export.

	(1)	(2)	(3)
*lnDIG*	1.676***		
	(0.498)		
*lnDIG_lag1*		1.2004^***^	
		(0.2624)	
*lnDIG_lag2*			1.1563^**^
			(0.2722)
*lnGDP*	0.710***	0.0917^***^	0.0976^***^
	(0.110)	(0.0233)	(0.0282)
*lnPOP*	0.0392*	0.0958	0.1406
	(0.070)	(0.1811)	(0.2058)
*lnDIS*	0.446	−0.0216	−0.0188
	(0.264)	(0.0267)	(0.0249)
*lnEDI*	0.157	0.0187	0.0157
	(0.208)	(0.0131)	(0.0124)
*lnBA*	0.114*	0.0117*	0.0046
	(0.089)	(0.0105)	(0.0188)
*lnINTER*	0.0476	0.0577	0.0707
	(0.0505)	(0.0475)	(0.0449)
Constant	−17.73***	−3.3410	−4.1249
	(2.185)	(3.0212)	(3.4134)
Country fixed effects		Yes	Yes
Time fixed effects		Yes	Yes
N	945	944	943
r^2		0.6693	0.6693

Note: Robust standard errors, clustered at the country level, are reported in parentheses. *p < 0.1, **p < 0.05, ***p < 0.01.

### 3.4 Heterogeneity analysis

(1) National Heterogeneity Analysis

The significant disparities in economic development levels across countries result in heterogeneous impacts on China’s digital service trade exports. To investigate these differences, we classified the income levels of 105 countries based on the World Bank’s criteria. Due to the limited number of low-income countries, they were included in the empirical regression of low and middle-income countries to avoid serious multicollinearity issues. The regression results of this classification are presented in **Table 3**.

**Table 6** illustrates that the development of the digital economy significantly promotes China’s digital service trade exports across all income levels at the 1% significance level. The coefficients for digital economy development in low and middle-income, middle-low + low-income, middle-and-high income, and high-income countries are 1.1303, 1.1544, 1.2656, and 1.2388, respectively. The largest coefficient, 1.2656, suggests that digital economy development in middle-and-high income countries has the most substantial impact on China’s digital service trade exports. This might be due to their rapid digital economy growth, advanced digital infrastructure, and substantial market potential [30,36]. With a coefficient of 1.2388***, high-income countries also play a crucial role, although their markets might be more saturated compared to middle-and-high income countries.

**Table 6:** Heterogeneity analysis across income-level country groups dependent variable: China’s Digital Service Export.

	Low and Middle Income (1)	Middle-Low + Low Income (2)	Middle-and-High Income (3)	High Income (4)
${lnDIG}_{it\;}$	1.1303**	1.1544**	1.2656***	1.2388***
	(0.3091)	(0.3088)	(0.2717)	(0.1895)
${lnGDP}_{it}$	0.6629***	0.6393***	0.7144***	0.4701***
	(0.0999)	(0.0891)	(0.0714)	(0.0491)
${lnPOP}_{it}$	−0.3203	−0.2446	−0.2427	0.5351^**^
	(0.3828)	(0.3320)	(0.4162)	(0.2639)
${lnDIS}_{i}$	−0.3207	−0.4154	−0.4910*	−0.4152*
	(0.3862)	(0.3921)	(0.2664)	(0.2367)
${lnEID}_{it}$	0.0264	0.0428	0.0358	0.0079
	(0.0309)	(0.0315)	(0.0249)	(0.0257)
${lnBA}_{it}$	0.0184*	0.0198	0.0245**	−0.0142*
	(0.0123)	(0.0291)	(0.0080)	(0.0186)
${lnINTER}_{it}$	0.0500	0.0366	0.0589^*^	0.0719
	(0.0578)	(0.0466)	(0.0346)	(0.0448)
_cons	3.5416	2.1761	1.4823	−9.3670^**^
	(7.2901)	(6.2780)	(6.5780)	(4.1247)
Country fixed effects	Yes	Yes	Yes	Yes
Time fixed effects	Yes	Yes	Yes	Yes
N	216	243	279	414
r^2_overall	0.5146	0.5101	0.4578	0.4559

Note: Robust standard errors, clustered at the country level, are reported in parentheses. *p < 0.1, **p < 0.05, ***p < 0.01.

(2) Industry Heterogeneity Analysis

In line with the classification criteria of the United Nations Conference on Trade and Development (UNCTAD), digital service trade is divided into six sub-sectors. This analysis delves into the impact of digital economy development on these sub-sectors to uncover sector-specific effects.

**Table 7** reveals that digital economy development significantly promotes all six sub-sectors of digital service trade at the 1% significance level. The coefficients for each sub-sector are as follows: With the highest coefficient (6.1357***), intellectual property services benefits the most from digital economy development. This implies that a 1% increase in digital economy development in trading countries boosts China’s intellectual property service exports by 6.1357%. The strong impact underscores the importance of an intellectual property framework in supporting digital economic elements like digital technology and data rights [40]. The second highest coefficient (2.7427***) indicates that a 1% increase in the digital economy of the importing country enhances China’s export of personal, cultural, and entertainment services by 2.7427%. This suggests that cultural export initiatives, such as establishing cultural export bases and promoting Chinese programs in international media, significantly contribute to this sector’s growth. A coefficient of 2.2372*** highlights the substantial positive effect of digital economy development on telecommunications, computer, and information Services. Improved digital infrastructure and technology adoption in trading countries drive the demand for these services. With a coefficient of 1.6281***, insurance and pension services also benefits considerably from digital economy growth. Enhanced digital financial systems and services likely contribute to this positive impact. A coefficient of 1.5756*** indicates that the digital economy also positively influences other business services, encompassing a range of professional services. Although the coefficient (1.1413***) is lower compared to other sub-sectors, financial services still signifies a robust positive relationship between digital economy development and the export of financial services. The consistent positive and significant coefficients across all sub-sectors reinforce the pivotal role of digital economy development in bolstering China’s digital service trade. The results suggest that strategic investments in digital infrastructure and technology, coupled with robust intellectual property frameworks and cultural export initiatives, are crucial for maximizing the benefits of digital service trade across various industries.

**Table 7:** Industry heterogeneity test results dependent variable: China’s Digital Service Export.

	Insurance and Pension Services (1)	Financial Services (2)	Intellectual Property Services (3)	Telecommunications, Computer and Information Services (4)	Personal, Cultural and Entertainment Services (5)	Other Business Services (6)
${lnDIG}_{it\;}$	1.6281***	1.1413***	6.1357***	2.2372***	2.7427***	1.5756***
	(0.2064)	(0.2937)	(0.4040)	2.2372***	(0.4123)	(0.1424)
_cons	4.6973	3.2956	−77.8582^**^	−6.0345	0.3091	−14.9749
	(10.3487)	(29.9110)	(30.8041)	(29.1790)	(0.5583)	(18.8953)
Variable of control	Yes	Yes	Yes	Yes	Yes	Yes
Country fixed effects	Yes	Yes	Yes	Yes	Yes	Yes
Time fixed effects	Yes	Yes	Yes	Yes	Yes	Yes
N	927	936	936	936	936	936
r^2_overall	0.8994	0.9211	0.9145	0.9727	0.9399	0.9883

Note: Robust standard errors, clustered at the country level, are reported in parentheses. *p < 0.1, **p < 0.05, ***p < 0.01.

## 4. Conclusions

This study extends the measurement framework of digital service trade by applying a gravity model to examine how digital economy development and related infrastructure shape China’s digital service exports. Our analysis yields several key insights. First, higher levels of digital economy development in partner countries significantly promote China’s digital service exports. Second, geographical distance continues to exert a negative influence, whereas institutional and economic system similarities enhance bilateral digital trade. Third, improvements in broadband penetration and internet adoption in partner economies are strongly associated with increased exports from China. Heterogeneity analysis reveals that the digital economy’s positive effects are more pronounced in middle- and high-income countries, likely due to their advanced digital infrastructure and market readiness. Sector-specific findings highlight intellectual property services as the leading beneficiary, followed by personal and cultural services, telecommunications, and financial services.

These results align with prior work [11,14] affirming the spillover benefits of digital infrastructure on global trade. Notably, we observe a broader sectoral impact than suggested by earlier studies [41]—based on empirical evidence from Kuzovkova and Tkachenko (2019) and Guo (2023), these earlier studies indicated that the digital economy predominantly benefits intellectual property, telecommunications, and financial services, with limited measurable impact on cultural and entertainment sectors. By contrast, our findings reveal broader effects, potentially attributable to the rapid expansion of China’s tourism and cultural industries in recent years; this growth, alongside rising global interest in Chinese culture, may have spurred international demand for digitally mediated tourism and entertainment services, thereby intensifying cross-border activity in these domains. This pattern potentially reflects China’s rising global profile in cultural and tourism services. The findings underscore the strategic value of international digital cooperation. China can deepen digital trade ties by supporting infrastructure development in partner countries and reducing distance-related barriers through regulatory and policy alignment.

This study have important policy implications. China should prioritize the expansion of domestic digital infrastructure, promote global digital governance collaboration, and enhance the international competitiveness of its digital service platforms. Differentiated strategies are essential to unlock the full potential of digital service trade across income levels and industry sectors, with particular emphasis on fostering intellectual property services as a core engine of growth.

This study focuses on macro-level patterns in China’s digital service exports, but it does not account for regional variation due to limited availability and quality of subnational data. Although initial attempts were made to explore geographic clustering—particularly in provinces like Zhejiang, home to major hubs such as Hangzhou—data gaps and inconsistencies prevented reliable empirical analysis. Future research could address this limitation by leveraging improved regional data to examine spatial clustering and the impact of province-specific policies, thereby strengthening identification strategies and offering deeper insights into the geography of digital service trade.

## Supporting information

S1 File Appendix. (DOCX)

S2 Data Data files. The dataset comprises the classification of digital economy development levels, country classifications, industry classifications, the digital economy development index, and digital service trade export volumes. (ZIP)
