# Supplementary material for: Leveraging the digital economy for enhanced digital service trade exports: Lessons from China
Source: PLoS One. 2025 Oct 30;20(10):e0333798. doi: 10.1371/journal.pone.0333798 (PMC12574928; doi:10.1371/journal.pone.0333798)
Supplement: S1 File — (DOCX) [file pone.0333798.s001.docx]

**Appendix**

1. **Appendix A**

(1) Correlation Test

Prior to the regression analysis, we assessed the potential collinearity among variables using Pearson’s correlation test. **Table A1** reports the pairwise correlations among the dependent variable, key explanatory variables, and controls.

The level of digital economy development exhibits a significant positive correlation with the export of digital services (r = 0.555, ***p < 0.01), suggesting that more advanced digital economies are associated with greater digital service exports from China. GDP shows a strong positive correlation with exports (r = 0.871, ***p < 0.01), consistent with the expectation that larger economies are more active in digital service trade. Population size is also positively correlated with exports (r = 0.235, ***p < 0.01), although its effect is more modest relative to GDP and per capita GDP (PGDP). Among control variables, economic institutional development (EID), broadband access (BA), and internet penetration (INTER) all correlate positively and significantly with export volumes, underscoring the importance of digital infrastructure and governance in promoting digital service trade. In contrast, geographical distance (DIS) is negatively correlated with exports and several explanatory variables, highlighting the persistent dampening effect of spatial separation on digital service trade.

**Table A1** Pearson Correlation Coefficients among Variables

|  | *EX* | *DIG* | *GDP* | *POP* | *DIS* | *EID* | *BA* | *INTER* |
| --- | --- | --- | --- | --- | --- | --- | --- | --- |
| *EX* | 1 |  |  |  |  |  |  |  |
| *DIG* | 0.555*** | 1 |  |  |  |  |  |  |
| *GDP* | 0.871*** | 0.416*** | 1 |  |  |  |  |  |
| *POP* | 0.235*** | 0.143*** | 0.327*** | 1 |  |  |  |  |
| *DIS* | -0.149*** | -0.280*** | -0.02 | -0.149*** | 1 |  |  |  |
| *EID* | 0.158*** | 0.305*** | 0.139*** | 0.046 | 0.082** | 1 |  |  |
| *BA* | 0.360*** | 0.807*** | 0.244*** | -0.159*** | -0.193*** | 0.353*** | 1 |  |
| *INTER* | 0.296*** | 0.769*** | 0.173*** | 0.204*** | -0.151*** | 0.291*** | 0.808*** | 1 |

Note: Robust standard errors, clustered at the country level, are reported in parentheses. *p<0.1, **p<0.05, ***p<0.01.

1. Multicollinearity Test

To assess the robustness of the model and mitigate potential bias from multicollinearity, we conducted a Variance Inflation Factor (VIF) analysis. VIF quantifies how much the variance of a regression coefficient is inflated due to correlation with other predictors. The results are presented in **Table A2**.

The average VIF across all variables is 6.47, below the commonly accepted threshold of 10, indicating that multicollinearity is not a major concern. The highest VIF is observed for *lnGDP* (13.65), suggesting moderate collinearity. *lnPOP* also exhibits a VIF slightly above 10, but still within an acceptable range. *lnDIG, lnBA*, and *lnINTER* have moderate VIFs, indicating tolerable levels of collinearity. *lnEID* and *lnDIS* show the lowest VIF values, reflecting minimal collinearity with other regressors. The corresponding 1/VIF values support these interpretations, with lower values signaling higher collinearity and values closer to one suggesting low redundancy among variables.

**Table A2** Variance inflation factors of explanatory variables

|  | VIF | 1/VIF |
| --- | --- | --- |
| ${lnGDP}_{it}$ | 13.65 | 0.0732 |
| ${lnPOP}_{it}$ | 10.29 | 0.0972 |
| ${lnDIG}_{it}$ | 6.12 | 0.163 |
| ${lnBA}_{it}$ | 4.36 | 0.229 |
| ${lnINTER}_{it}$ | 3.24 | 0.308 |
| ${lnEID}_{it}$ | 1.27 | 0.784 |
| ${lnDIS}_{it}$ | 1.20 | 0.833 |
| Mean VIF | 6.47 | |

1. **Appendix B**

Given the use of panel data, three standard estimation approaches were considered: pooled ordinary least squares (OLS), random effects, and fixed effects models. Model selection was guided by a sequence of diagnostic tests, including the F-test, Lagrange Multiplier (LM) test, and Hausman test, with results summarized in **Table B1**.

The F-test rejected the null hypothesis that the pooled OLS model suffices, indicating that the fixed effects model offers a statistically significant improvement in explanatory power. The LM test, applied to assess the suitability of the random effects model over pooled OLS, also favoured a panel structure. However, the Hausman test did not reject the null hypothesis of no systematic difference between the fixed and random effects estimators, suggesting that the random effects specification is appropriate under the assumption of orthogonality between regressors and unobserved individual heterogeneity.

**Table B1** Model selection test results

|  | F test | | LM test | | Hausman test | |
| --- | --- | --- | --- | --- | --- | --- |
| ${lnEX}_{it}$ | Statistic of F | P value | Value of statistics | P value | $\chi^{2}$ | P value |
|  | 320.80 | 0.000 | 1331.55 | 0.090 | 8.47 | 0.132 |

We evaluate the estimation results derived from the three panel regression models, as presented in **Table B2**. Across all model specifications, the core explanatory variable exerts a statistically significant and positive influence on the dependent variable. This consistency underscores the robustness of the association to alternative methodological choices. As expected, time-invariant variables—such as geographic distance—are omitted under fixed effects estimation due to the within-group transformation inherent in the model. The estimated coefficients of other covariates remain broadly stable across specifications, further supporting the reliability of the findings.

**Table B2** Regression analysis of panel data models

|  | Mixed effects model | Random effects model | Fixed effects model |
| --- | --- | --- | --- |
| ${lnDIG}_{it}$ | 1.123*** | 2.050*** | 2.103*** |
|  | (0.161) | (0.112) | (0.115) |
| ${lnGDP}_{it}$ | 0.747*** | 0.568*** | 0.504*** |
|  | (0.026) | (0.038) | (0.051) |
| ${lnPOP}_{it}$ | 0.1224** | 0.0910*** | 0.0886*** |
|  | （0.1931） | (0.0100) | (0.0091) |
| ${lnDIS}_{i}$ | 0.510*** | 0.467*** | 0.000 |
|  | (0.059) | (0.168) | (.) |
| ${lnEID}_{it}$ | 0.082 | 0.114** | 0.120** |
|  | (0.086) | (0.047) | (0.047) |
| ${lnBA}_{it}$ | 0.057 | 0.080*** | 0.076** |
|  | (0.037) | (0.029) | (0.031) |
| ${lnINTER}_{it}$ | -0.450*** | -0.223*** | -0.215*** |
|  | (0.091) | (0.048) | (0.050) |
| Constant | -15.717*** | -14.259*** | -16.998*** |
|  | (0.794) | (1.849) | (1.255) |
| N | 945 | 945 | 945 |
| r^2_overall | 0.810 | 0.801 | 0.783 |

Note: Robust standard errors, clustered at the country level, are reported in parentheses. *p<0.1, **p<0.05, ***p<0.01.

The regression results of the random effects model show that among the control variables, the gross domestic product (${lnGDP}_{jt}$) of the trading partner has a significantly positive impact on China's digital service trade, indicating that the GDP of the trading partner significantly promotes China's service trade exports. The coefficient of geographical distance (${lnDIS}_{ij}$) has a negative impact on China's digital service trade exports at the 1% significance level, with a coefficient value of -0.467. Economic institutional distance (${lnEID}_{ijt}$) significantly promotes the export of digital service trade, suggesting that there is a "learning possibility" when countries engage in digital trade activities. Countries with relatively backward economic institutional environments learn from those with a comparative advantage in economic institutions, thereby optimizing their market economic environment and digital technology infrastructure related to digital service trade. The number of fixed broadband users in the trading partner (FBU) has a negative impact on China's digital service trade exports at the 1% significance level, with a coefficient value of -0.467. This indicates that an increase in the number of broadband users in the trading partner may drive the rise of its domestic digital service industry, creating a competitive situation with Chinese enterprises in the international market and squeezing the export space of China's digital service trade. At the same time, an increase in the number of users changes the local demand structure for digital services, making consumers more inclined to choose local services and reducing the demand for Chinese digital services. In addition, the improvement of broadband infrastructure in the trading partner may also prompt it to introduce protective policies or trade barriers, further hindering China's digital service exports. Higher levels of Internet development in partner countries are associated with greater digital service exports from China. This may reflect how widespread Internet access stimulates demand for network-based services across enterprises, governments and households, thereby increasing imports of digital services trade [1].

1. **Appendix C**

The Global Digital Economy Development Index (TIMG) is a composite measure developed by the Institute of Finance and Banking at the Chinese Academy of Social Sciences, the National Laboratory of Finance and Development, and affiliated institutions. Details on its compilation methodology can be found in Wang et al. (2021)[2]. The index assesses the digital economy performance of major global economies across four dimensions: digital technology, digital infrastructure, digital markets, and digital governance. It covers 106 economies from 2013 to 2021. **Table C1** reports the digital economy development levels for selected trading partner countries.

**Table C1** Digital economy development index for selected trading partner countries

| Country | Year | TIMG index | Index of Components | | | |
| --- | --- | --- | --- | --- | --- | --- |
|  |  |  | Digital Technology Index | Digital Infrastructure Index | Digital Market Index | Digital Governance Index |
| Albania | 2021 | 45.71 | 39.91 | 53.34 | 34.89 | 54.7 |
| Algeria | 2021 | 41.12 | 37.74 | 46.86 | 41.48 | 38.4 |
| Argentina | 2021 | 56.98 | 45.54 | 57 | 69.03 | 56.36 |
| Armenia | 2021 | 46.73 | 38.69 | 48.44 | 35.18 | 64.62 |
| Australia | 2021 | 79.73 | 71.99 | 84.5 | 76.57 | 85.87 |
| Austria | 2021 | 75.88 | 71.47 | 77.53 | 70.04 | 84.5 |
| Azerbaijan | 2021 | 55.42 | 46.86 | 67.35 | 34.8 | 72.69 |
| Bahrain | 2021 | 59.55 | 43.04 | 61.75 | 55.27 | 78.15 |
| Bangladesh | 2021 | 45.3 | 27.69 | 72.39 | 46.87 | 34.24 |
| Belgium | 2021 | 76.5 | 74.6 | 79.87 | 75.55 | 75.97 |
| Bolivia | 2021 | 30.52 | 20.75 | 37.24 | 30.25 | 33.86 |
| Botswana | 2021 | 37.48 | 28.1 | 51.61 | 23.22 | 46.97 |
| Brazil | 2021 | 66.77 | 44.99 | 84.74 | 82.12 | 55.21 |
| Bulgaria | 2021 | 58.24 | 46.45 | 63.95 | 57.87 | 64.7 |
| Cameroon | 2021 | 35.67 | 34.03 | 47.48 | 27.09 | 34.08 |
| Canada | 2021 | 80.65 | 74.69 | 82.89 | 84.97 | 80.06 |
| Chile | 2021 | 62.68 | 49.19 | 65.11 | 65.15 | 71.27 |
| China | 2021 | 81.42 | 74.17 | 89.33 | 95.57 | 66.61 |
| Colombia | 2021 | 57.3 | 42.23 | 63.75 | 64.96 | 58.25 |
| ... | ... | ... | ... | ... | ... | ... |
| Turkey | 2013 | 55.85 | 51.96 | 59.97 | 58.99 | 52.46 |
| Uganda | 2013 | 27.87 | 21.24 | 42.72 | 16.93 | 30.6 |
| Ukraine | 2013 | 44.13 | 48.44 | 48.02 | 45.99 | 34.07 |
| United Arab Emirates | 2013 | 58.31 | 53.82 | 46.97 | 55.63 | 76.83 |
| United Kingdom | 2013 | 78.85 | 72.7 | 71.2 | 83.57 | 87.93 |
| United States | 2013 | 86.41 | 87.06 | 79.79 | 94.99 | 83.81 |
| Uruguay | 2013 | 44.28 | 32.03 | 52.34 | 37.01 | 55.76 |
| Venezuela, RB | 2013 | 29.25 | 26.78 | 37.84 | 23.5 | 28.87 |
| Vietnam | 2013 | 39.64 | 31.66 | 48.39 | 37.26 | 41.24 |
| Yemen, Rep | 2013 | 14.25 | 9.13 | 22.3 | 13.57 | 12.02 |
| Zimbabwe | 2013 | 18.71 | 20.65 | 20.69 | 8.15 | 25.35 |

**References**

1. Wu, D., and Y. Wu. 2020. Influence of Trade Facilitation on China’s Imports From “The Belt and Ｒoad”Countries. Journal of Industrial Technological Economics 39(2): 73-81.
2. Wang, Zhe, Chen, Yinmo, and Zhang, Ming. 2021. Measuring Global Digital Economy Development: The TIMG Index and New Stylized Facts. Financial Review (6): 40-56.
